# Hypodontia of mandibular incisors: considerations on the orthodontic treatment

**DOI:** 10.1590/2177-6709.23.4.079-087.bbo

**Published:** 2018

**Authors:** Renato Barcellos Rédua, Paulo César Barbosa Rédua

**Affiliations:** 1 Escola Superior São Francisco de Assis, Faculdade de Odontologia, Disciplina de Clínica Integrada Infantil (Santa Tereza/ES, Brazil). Escola Superior São Francisco de Assis Escola Superior São Francisco de Assis Faculdade de Odontologia Santa TerezaES Brazil; 2 Private practice (Vitória/ES, Brazil). VitóriaES Brazil

**Keywords:** Agenesis, Hypodontia, Orthodontic space closure, Tooth movement techniques

## Abstract

Hypodontia is the most prevalent craniofacial malformation in mankind. It may present a wide variety of manifestations and, depending on the number and location of missing teeth, it may affect the esthetics, mastication, speech and occlusal balance. This paper discusses the therapeutic approaches to solve this condition, describing a case report with hypodontia of one mandibular lateral incisor, which treatment option included space closure at the region of hypodontia associated with composite resin restorations in the mandibular central incisors. The three-year follow-up after treatment revealed occlusal stability, adequate intercuspation in Class I relationship and excellent micro and macroesthetics.

## Resumo

A agenesia dentária é a malformação craniofacial mais prevalente em humanos. Pode apresentar uma grande variedade de manifestações e, dependendo do número e localização dos dentes ausentes, pode afetar a estética, a mastigação, a fala e o equilíbrio da oclusão. Neste artigo, serão discutidas as propostas terapêuticas para resolução dessa condição e será descrito um caso clínico com agenesia de um incisivo lateral inferior, cuja opção de tratamento foi o fechamento do espaço remanescente da agenesia, associado ao incremento de resina composta nos incisivos centrais inferiores. No acompanhamento por três anos após o tratamento, verificou-se estabilidade oclusal, adequada intercuspidação em Classe I e excelente micro e macroestética.

## INTRODUCTION

Hypodontia, or tooth agenesis, is the lack of formation of one or more teeth, being the most prevalent craniofacial malformation in mankind; it may occur as part of a known genetic syndrome or as a non-syndromic isolated trait.^1^

It presents a wide variety of manifestations and, depending on the number and location of missing teeth, it may affect the esthetics, mastication, speech and occlusal balance, due to undesirable occlusal contacts caused by the extrusion of antagonist teeth or inclination of teeth adjacent to the affected area,^2^ with impact on the quality of life of affected individuals.^3^

Tooth agenesis is classified according to the number of non-formed teeth, except for the third molars. Hypodontia is the term employed to indicate agenesis of one to five teeth; oligodontia, when there are six or more congenitally missing teeth; and anodontia refers to the complete absence of tooth formation.^1^

Except for the third molars, the reported prevalence of hypodontia ranges from 1.6 to 6.9%,^1^ while it is very rare in deciduous teeth.^4^ Polder et al^5^ conducted a meta-analysis and observed higher prevalence of non-syndromic tooth agenesis in Europe (4.6% men, 5.5% women) and Australia (5.5% men, 7.6% women) compared to Caucasians in North America (3.2% men and 4.6% women). The most affected teeth include the mandibular second premolars and maxillary lateral incisors, followed by the mandibular incisors and maxillary premolars.^5^ The mandibular second premolars are the most affected teeth in individuals from both European and Asian descent, followed by the permanent maxillary lateral incisors or maxillary second premolars, in the European population^6^, and the mandibular and maxillary incisors and maxillary second premolars in Asian individuals.^7^

The prevalence of tooth agenesis seems to be similar in the maxilla and mandible^1^^,^^5^ and is unilateral in most cases.^1^^,^^8^ Comparison of bilateral and unilateral hypodontia of the maxillary lateral incisors demonstrated that the bilateral occurrence was more frequent than the unilateral, while unilateral agenesis was more common in the other teeth.^5^

The hereditary component is an important causal factor,^9^ and studies demonstrate concordant frequency of this condition in siblings.^10^

The literature is controversial regarding the prevalence of this condition between genders, even though the permanent dentition seems to reveal a slight, non-significant predilection of hypodontia in females.^1^^,^^11^ However, significant difference has also been observed in females, with prevalence 1.4 times higher than in males.^5^

The literature suggests an increase in the prevalence of hypodontia in the last decades;^12^ however, there are no evidences to support whether this apparent increase is related to more advanced methods of screening and diagnosis or to other factors.^1^

This condition is frequently non-syndromic, yet it may be associated with cleft lip and/or palate^13^ and several other syndromes, including Down syndrome and ectodermal dysplasia.^1^ Recent data also suggest that hypodontia shares some common aspects with certain types of cancer.^14^

It is not known whether individuals with hypodontia have distinguished skeletal characteristics, even though some studies suggest that individuals with hypodontia present different craniofacial features than individuals without missing teeth.^1^ It is known that tooth agenesis, especially the most severe manifestations, contributes to the abnormal occlusion and is frequently associated with anomalies in other teeth, such as alterations of shape, especially peg-shaped teeth or microdontia.^1^^,^^2^ Other common characteristic of hypodontia is the ectopic positioning of adjacent permanent teeth, due to the absence of neighboring teeth to guide the eruption or due to lack of space for eruption.^1^

The treatment of hypodontia varies according to its complexity, being more critical in young and growing patients, impairing both the psychological aspect and facial development of these individuals, requiring multidisciplinary treatment.^2^^,^^15^^,^^16^ Even though the agenesis of mandibular incisors is not among the most frequent manifestations of hypodontia,^1^^,^^8^ it represents a complex clinical challenge, since in most cases there is loss of the deciduous tooth, impairing the function and occlusal balance, often leading to deep bite, residual overjet, mandibular midline deviation and positive tooth-size discrepancy due to the tooth absence.^10^

The therapeutic options described in the literature for growing patients include space maintenance with fixed or removable space retainers, with or without fixed denture,^10^ until completion of growth, followed by placement of the definitive implant.^17^ In adult patients, it is possible to perform orthodontic treatment for implant placement or to close the remaining space, finalizing with three mandibular incisors.^18^ This treatment option leads to large tooth-size discrepancy between the dental arches,^19^ which may also be present in approximately 60% of orthodontic patients, besides the cases of tooth agenesis.^20^^,^^21^^,^^22^

This paper discusses the therapeutic options for resolution of tooth agenesis and reports the orthodontic treatment of a growing patient with hypodontia of one mandibular lateral incisor. 

## CASE REPORT

**Figure 1:**
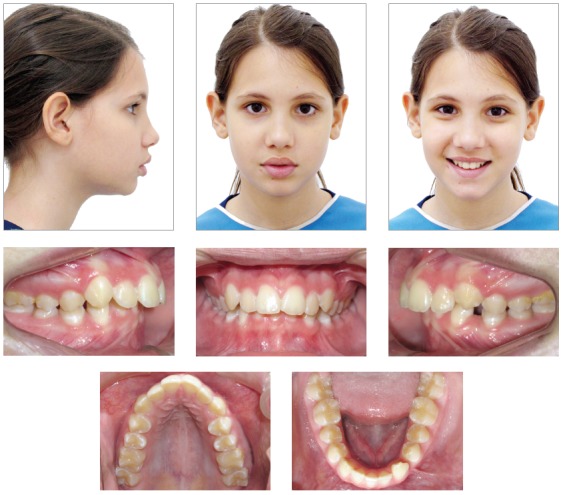
Initial facial and intraoral photographs.

Female patient aged 11 years and 2 months, previously diagnosed with agenesis of tooth #32, was referred by the pediatric dentist for orthodontic evaluation. The patient reported dissatisfaction with *“misaligned lower teeth and very narrow smile”* (Fig 1). The medical and dental histories were uneventful, and anamnesis revealed no familial occurrence of hypodontia.

The facial analysis evidenced symmetry in frontal view, straight nasolabial angle, lack of lip seal, everted lower lip, balanced dimensions of the lower facial third and convex profile (Fig 1). The functional analysis demonstrated adequate exposure of maxillary incisors during speech and smile. There were no sounds or symptoms of temporomandibular disorder, nor deviations in mandibular movements. 

**Figure 2:**
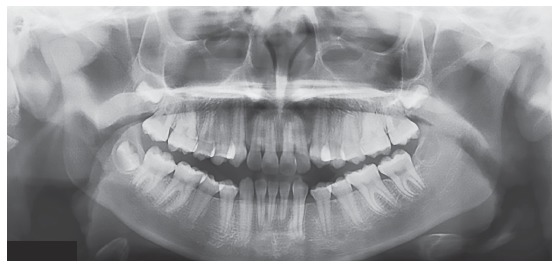
Initial panoramic radiograph.

The occlusal analysis revealed that the patient was in the permanent dentition stage, with absence of tooth #32 confirmed by panoramic radiograph (Fig 2). The patient presented Angle Class II division 1 malocclusion with 8-mm overjet, deep bite, coincident maxillary and mandibular midlines, positive tooth-size discrepancy of 5 mm in the mandibular arch and negative of 2 mm in the maxillary arch, and slight constriction at the region of maxillary premolars. The patient presented good oral hygiene without restorations or carious lesions (Fig 1).

**Figure 3:**
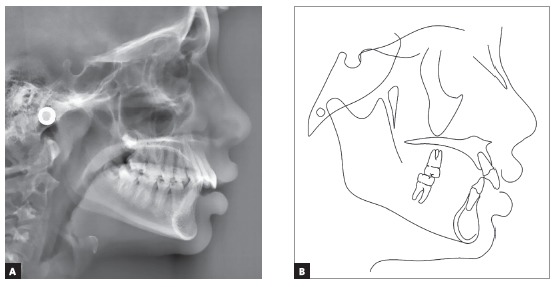
Initial lateral cephalogram (A) and cephalometric tracing (B).

Cephalometric analysis confirmed the skeletal Class II pattern with ANB of 5^o^, convexity angle of 7^o^, adequate mandibular plane and adequate axial inclinations of maxillary and mandibular incisors. Analysis of maturation of cervical vertebrae indicated that the patient could be on onset of the pubertal growth spurt (Fig 3).

## TREATMENT PLANNING AND MECHANICS EMPLOYED

The treatment goals were to enhance the smile esthetics with good function and occlusal stability. To correct the Class II and change the facial growth pattern, the treatment included cervical headgear used 14h per day, with approximate force of 400g per side,^23^ which would also reduce the overjet and allow passive lip seal. 

Fixed Edgewise straight-wire metallic appliances (Roth prescription), with 0.022 x 0.028-in slot, were used for tooth leveling, to correct the curve of Spee and deep bite, and for space closure in the mandibular arch, finalizing with three incisors. To correct the tooth-size discrepancy,^20^^,^^21^ it was decided to directly apply composite resin on the proximal surfaces of both mandibular central incisors, avoiding proximal stripping on the maxillary incisors that presented optimal proportion between height and width, to achieve an ideal esthetics of smile with predominance of the maxillary incisors.^24^

Tooth alignment and leveling were performed with 0.014-in nickel-titanium archwires and 0.016 to 0.020-in stainless steel archwires for nine months. After distalization of maxillary canines, 0.019 x 0.025-in stainless steel archwires were placed with Class II intermaxillary elastics associated with the headgear.

After achievement of intercuspation of molars, premolars and canines in Class I, a 3-mm diastema was present between teeth #33 and #31. At this moment, tooth #31 was distalized and received 0.8 mm of composite resin on the mesial surface and 0.7 mm on the distal surface. Thereafter, tooth #41 was also distalized and received 0.7 mm of composite resin on the mesial surface and 0.8 mm on the distal surface. The composite resins were adapted by the orthodontist, without removal of brackets; thus, at the end of treatment, requiring replacement by a specialized professional.

After treatment completion, a 3 x 3 mandibular retainer fabricated with 0.028-in stainless steel archwire was bonded to teeth #33 and #43, and a maxillary retainer fabricated with 0.018-in stainless steel archwire was bonded to teeth #11 and #21, both for undetermined period.^25^ A removable maxillary wraparound retainer was also fabricated for full-time utilization during the first 6 months and nighttime use for further 6 months.^26^ The overall period of active treatment was 30 months, and examinations were requested to evaluate the outcomes.

## TREATMENT OUTCOMES

**Figure 4:**
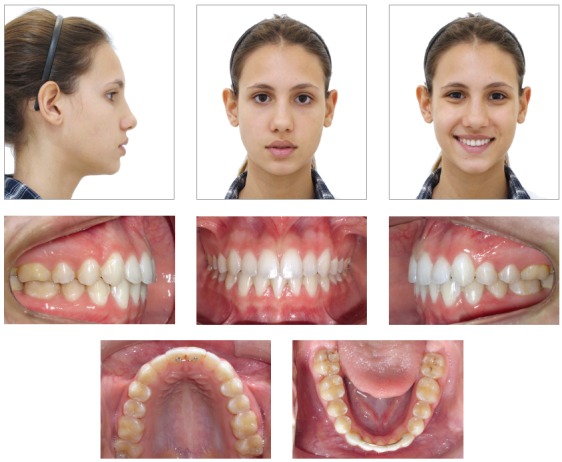
Final facial and intraoral photographs.

**Figure 5:**
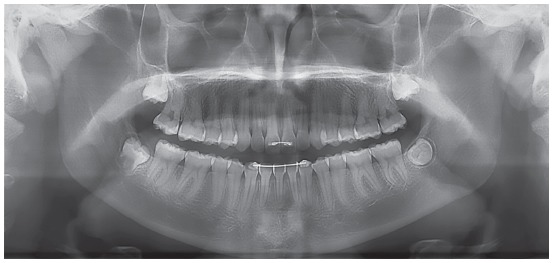
Final panoramic radiograph.

**Figure 6:**
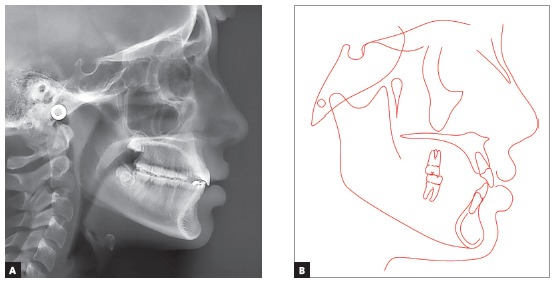
Final lateral cephalogram (A) and cephalometric tracing (B).

**Figure 7:**
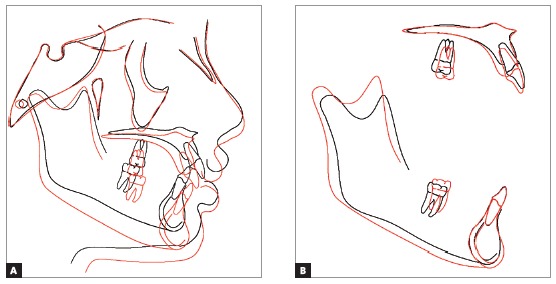
Total (A) and partial (B) superimpositions of initial (black) and final (red) cephalometric tracings.

The treatment objectives were achieved, namely Angle Class I molar relationship, adequate intercuspation of premolars and canines, canine guidance, adequate contact points, overbite of 2 mm and ideal overjet with mutually protected anterior guidance, despite the finalization with three mandibular incisors. Also, the maxillary midline was coincident with the facial midline and centralized in relation to the mandibular incisor in central position. The patient also presented good periodontal health, with ideal gingival contour, following the concepts of balanced ideal occlusion, with balanced distribution of forces in vertical, lateral and sagittal directions^27^ and achieving the concepts of smile esthetics (Figs 4 and 5).^24^ A straight profile was achieved and the lip relationship was improved, with passive lip seal at treatment completion, attained by the changes in facial growth pattern, with reduction of ANB achieved by mandibular growth and orthopedic restriction of maxillary growth by utilization of the cervical headgear (Figs 6 and 7, and Table 1).^28^

**Table 1:** Initial (A) and final (B) cephalometric values.

	Measurements		Normal	A	B	A/B Dif.
Skeletal pattern	SNA	(Steiner)	82^o^	84^o^	81^o^	3
	SNB	(Steiner)	80^o^	79^o^	80^o^	1
	ANB	(Steiner)	2^o^	5^o^	1^o^	4
	Angle of convexity	(Downs)	0^o^	7^o^	0^o^	7
	Y-axis	(Downs)	59^o^	59^o^	59^o^	0
	Facial angle	(Downs)	87^o^	84^o^	85^o^	1
	SN-GoGn	(Steiner)	32^o^	27^o^	31^o^	4
	FMA	(Tweed)	25^o^	20^o^	20^o^	0
Dental pattern	IMPA	(Tweed)	90^o^	104^o^	102^o^	2
	1.NA (degrees)	(Steiner)	22^o^	26^o^	26^o^	0
	1-NA (mm)	(Steiner)	4 mm	6mm	6mm	0
	1.NB (degrees)	(Steiner)	25^o^	21^o^	25^o^	4
	1-NB (mm)	(Steiner)	4 mm	3mm	3mm	0
	- Interincisal angle	(Downs)	130^o^	128^o^	128^o^	0
	1-APo	(Ricketts)	1 mm	4mm	0mm	4
Profile	Upper lip - S-line	(Steiner)	0 mm	4mm	3mm	1
	Lower lip - S-line	(Steiner)	0 mm	4mm	3mm	1

**Figure 8:**
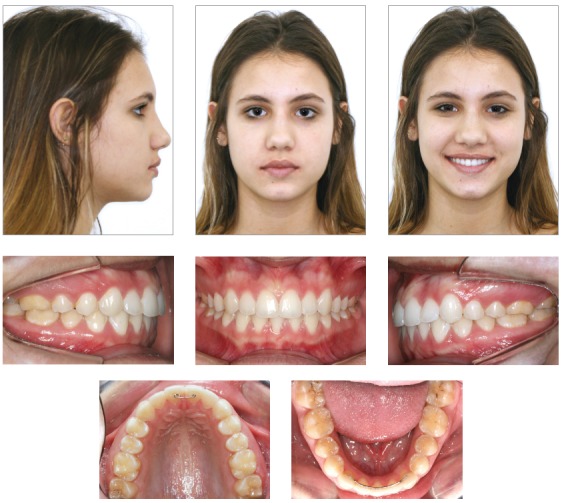
Facial and intraoral photographs three years after orthodontic treatment.

The evaluation three years after treatment completion revealed stability of outcomes achieved from both occlusal, functional and esthetic standpoints (Fig 8). 

## DISCUSSION

The safe and ideal orthodontic treatment planning for individuals with hypodontia of mandibular incisors requires the utilization of tools to diagnose the tooth-size discrepancies and/or discrepancies in ideal proportions between maxillary and mandibular teeth, to achieve a perfect intercuspation. The Bolton analysis is the most often used method for such evaluation.^20^^,^^21^ Though widely diffused and relatively simple to use, several professionals do not use it during clinical evaluation. According to Sheridan,^29^ only 47% of orthodontists interviewed by the author used this index frequently. Currently, with the advent of intraoral and dental cast scanners, this analysis may be digitally performed, using several softwares in a faster and simpler manner, with scientifically demonstrated accuracy.^30^^-^^32^

Besides the treatment option adopted in the present case, the hypodontia of one mandibular incisor may be managed by other treatment approaches: 1) maintenance of the three mandibular incisors, proximal stripping and retraction of maxillary incisors to correct the residual overjet resulting from space closure in the mandibular arch, thus minimizing the tooth-size discrepancy;^20^^,^^21^ or 2) space opening to insert an endosseous implant at the region of tooth #32. The main disadvantage of this treatment option is the need of implant placement, which may only be done after growth completion;^17^ thus, a provisional denture should be used at the implant region until surgery may be timely performed.

Analyzing these therapeutic options, we considered inadequate to maintain or achieve space for implant placement in a young individual, due to the need to wait for enough bone maturity to allow safe implant placement. Besides the esthetic discomfort, even with a provisional denture, there is mainly the disadvantage of alveolar bone loss at the region, with unfavorable prognosis for implant placement.^10^^,^^17^

Conversely, it should be considered that space closure at the region of the missing mandibular incisor leads to a tooth-size discrepancy between the dental arches,^19^ commonly clinically observed as a residual overjet, and occasionally by an increased overbite, even after achievement of ideal intercuspation of canines and premolars. However, the clinical alternatives to compensate this discrepancy and thus achieve ideal overjet and overbite are minimally invasive, either by placement of composite resin on the mandibular incisors, or by proximal stripping on the maxillary incisors.^18^^,^^19^

The decision to use composite resin on the mandibular incisors or proximal stripping on maxillary incisors, or the combination of both, should consider analysis of the morphology of maxillary and mandibular incisors. Maxillary central incisors with triangular buccal surface are more favorable for proximal stripping, as well as individuals whose buccal surface width is at least 75% of its height, or more. However, when the width of the buccal surface is smaller than 75% of its height, or when proximal stripping reaches this limit of proportion, the placement of composite resin on the mandibular incisors should be considered, thus maintaining an ideal proportion of height and width of the maxillary central incisors, which is fundamental to achieve the ideal microesthetics of smile.^24^

## CONCLUSION

The treatment of hypodontia of one mandibular incisor with maintenance of three incisors allows achievement of ideal intercuspation, overbite and overjet, long-term stability and smile esthetics, corroborating the ideal patterns of finalization and esthetics described in the literature.
